# Incidence and case fatality of stroke in Korea, 2011-2020

**DOI:** 10.4178/epih.e2024003

**Published:** 2023-12-26

**Authors:** Jenny Moon, Yeeun Seo, Hyeok-Hee Lee, Hokyou Lee, Fumie Kaneko, Sojung Shin, Eunji Kim, Kyu Sun Yum, Young Dae Kim, Jang-Hyun Baek, Hyeon Chang Kim

**Affiliations:** 1 Department of Public Health, Yonsei University Graduate School, Seoul, Korea; 2 Department of Preventive Medicine, Yonsei University College of Medicine, Seoul, Korea; 3 Department of Internal Medicine, Yonsei University College of Medicine, Seoul, Korea; 4 Institute for Innovation in Digital Healthcare, Yonsei University, Seoul, Korea; 5 Department of Neurology, Chungbuk National University Hospital, Cheongju, Korea; 6 Department of Neurology, Yonsei University College of Medicine, Seoul, Korea; 7 Department of Neurology, Kangbuk Samsung Hospital, Sungkyunkwan University School of Medicine, Seoul, Korea

**Keywords:** Stroke, Incidence, Case fatality rate

## Abstract

**OBJECTIVES:**

Stroke remains the second leading cause of death in Korea. This study was designed to estimate the crude, age-adjusted and age-specific incidence rates, as well as the case fatality rate of stroke, in Korea from 2011 to 2020.

**METHODS:**

We utilized data from the National Health Insurance Services from January 1, 2002 to December 31, 2020, to calculate incidence rates and 30-day and 1-year case fatality rates of stroke. Additionally, we determined sex and age-specific incidence rates and computed age-standardized incidence rates by direct standardization to the 2005 population.

**RESULTS:**

The crude incidence rate of stroke hovered around 200 (per 100,000 person-years) from 2011 to 2015, then surged to 218.4 in 2019, before marginally declining to 208.0 in 2020. Conversely, the age-standardized incidence rate consistently decreased by 25% between 2011 and 2020. When stratified by sex, the crude incidence rate increased between 2011 and 2019 for both sexes, followed by a decrease in 2020. Age-standardized incidence rates displayed a downward trend throughout the study period for both sexes. Across all age groups, the 30-day and 1-year case fatality rates of stroke consistently decreased from 2011 to 2019, only to increase in 2020.

**CONCLUSIONS:**

Despite a decrease in the age-standardized incidence rate, the total number of stroke events in Korea continues to rise due to the rapidly aging population. Moreover, 2020 witnessed a decrease in incidence but an increase in case fatality rates.

## GRAPHICAL ABSTRACT

[Fig f5-epih-46-e2024003]

## Key Message

This nationwide study using Korean National Health Insurance System data reveals a decade-long downward trend in overall stroke incidence rates. While the crude incidence rate showed a temporary increase from 2016 to 2019 before a slight decline in 2020, the age-standardized incidence rate consistently decreased over the study period. The study emphasizes the significance of continuous monitoring and preventive strategies to address stroke as a public health concern in Korea

## INTRODUCTION

According to the Global Burden of Diseases, Injuries, and Risk Factors Study, stroke is the second most common cause of death and the third most common cause of disability-adjusted life-years (DALYs) worldwide [1-3]. In Korea, stroke also stands as the second most common cause of death and DALYs, accounting for approximately 12% of all deaths and 7% of all DALYs in the country [4].

Over the past 2 decades, studies have been leveraging health insurance claims data, such as the Korea Medical Insurance Corporation study and the Korean National Health System Prospective Cohort study, to explore cardiovascular diseases and their risk factors. However, the primary aims of these studies are not to investigate the trends of cardiovascular diseases, with incidence rates often derived as secondary outcomes during the analysis process [5,6]. In 2020, the Korean Neurosurgical Society presented a noteworthy comprehensive analysis of stroke incidence trends in Korea from 2008 to 2016, utilizing national health insurance big data to analyze the trends surrounding stroke incidence in the country during that period [7,8].

The National Health Insurance System (NHIS) in Korea, due to its comprehensive coverage of the entire population, serves as an invaluable resource in offering insights into severe conditions such as stroke. However, while assessing stroke incidence trends, it is crucial to recognize the challenges introduced by divergent event-identification methods and data sources used in different studies. Nonetheless, exclusive reliance on claim diagnosis codes may not offer immediate or accurate estimations of incidence rates. Thus, the aim of this study was to estimate both the incidence and case fatality rates of acute stroke events in Korea over the period from 2011 to 2020 using a newly devised and reliable methodology based on claims data.

## MATERIALS AND METHODS

### Data source

We used the nationwide anonymized health information database of the NHIS, which is the single provider of mandatory health insurance, covering approximately 97% of the Korean population. Organized by the NHIS, the National Health Insurance (NHI) Big Data contains socio-demographics, hospital claims with International Classification of Diseases, 10th revision (ICD-10) coding, and fatality data of the entire population of Korea [9]. It is important to note that the fatality data included in our study are not directly linked to the records of the National Statistical Office.

### Ascertainment of stroke events

The data extraction was focused on patients admitted for stroke based on ICD-10 diagnosis codes (I60, I61, I63, I64) between January 1, 2002 and December 31, 2020. All relevant health insurance claim records were retrieved for these subjects. Health insurance claims data often associate multiple claims with a single disease event, necessitating the consolidation of various claim codes pertaining to drug prescriptions, diagnostic tests, and procedures. These codes can be scattered across multiple claims, complicating a comprehensive and accurate understanding of the disease episode. Consequently, we introduced the concept of a “hospitalization episode.” In short, 2 consecutive insurance claims, A and B, were considered separate hospitalization episodes under 2 conditions: (1) if the gap between the initial dates of claims A and B exceeded 28 days, and (2) if the interval between the final date of claim A and the initial date of claim B was 3 days or more. This method facilitated a more effective capture of the complete range of patient treatment and outcomes tied to each distinct hospitalization episode.

Each hospitalization episode served as the subject for stroke event identification. We developed specific identification algorithms for the first and recurrent stroke events, as detailed in Table 1; the detailed methods have been published elsewhere [10]. Although both algorithms primarily relied on the use of ICD-10 diagnosis codes (I60, I61, I63, I64), the algorithm for recurrent events included additional rigorous criteria. These diagnosis codes were supplemented with relevant diagnostic test and/or procedure codes.

To ensure the accuracy of these stroke identification algorithms, we conducted a retrospective review of medical records. This review included a total of 1,741 events from 24 hospitals throughout Korea, including 5 tertiary, 11 secondary, and 8 primary hospitals. We developed epidemiological adjudication criteria grounded on the 2013 American Heart Association/American Stroke Association definition of stroke [11], albeit with modifications, to determine the validity of each event identified by the algorithms. Positive predictive values (PPVs) were calculated by dividing the number of algorithm-identified events adjudicated as true acute stroke cases by the total number of algorithm-identified events examined. PPVs were calculated separately for first and recurrent events, as well as by hospital type (tertiary, secondary, and primary). The methodology for the design of these identification algorithms has been separately reported.

### Statistical analysis

We calculated both crude and age-standardized incidence rates of stroke (per 100,000 person-years) throughout the duration of the study period. Direct age-standardized rates were calculated using the 2005 Korean population to facilitate the comparison of yearly rates. To compare incidence rates between males and females, we calculated sex-specific incidence rates using similar methods, utilizing separate standard populations for each sex. Additionally, we calculated the case fatality rate of stroke in 2 forms: the 30-day fatality rate and the 1-year fatality rate. Across the period from 2011 to 2020, we computed both aggregate and age-stratified case fatality rates. The age groups were categorized into 40-64 years, 65-79 years, and 80 years or older. All statistical analyses were conducted using R version 4.0.3 (R Foundation for Statistical Computing, Vienna, Austria).

### Ethics statement

This study complied with the Declaration of Helsinki, and the study protocol was approved by the Institutional Review Board (IRB) of Yonsei University Health System, Seoul, Korea (#4-2022-0586). Informed consent was waived by the IRB. Informed consent was waived since this was a retrospective study of de-identified administrative data.

## RESULTS

### Number of stroke events, 2011-2020

Table 2 shows the number of identified stroke events during our study period. From 2011 to 2015, the total number of stroke events remained relatively consistent, with approximately 100,000 cases reported annually. From 2016 to 2019, there was a gradual increase in cases, before a minor decline to 106,807 cases in 2020. The trend in the number of first stroke events closely paralleled the trend in total stroke events. Conversely, the number of recurrent stroke events exhibited a steady rise from 2011 to 2019, followed by a slight decrease in 2020. The proportion of recurrent stroke among all stroke events also showed an upward trajectory, increasing from 16.6% in 2011 to 19.0% in 2020. When stratified by sex, the number of first stroke events in males increased from 2011 to 2019 but decreased in 2020, while the corresponding number in females remained relatively stable. As for the number of recurrent stroke events, both males and females experienced a substantial increase from 2011 to 2019, with decrease in 2020. Notably, the rate of increase in the number of stroke events was more pronounced in males compared to females, resulting in an increase in the male-to-female ratio from 1.1 in 2011 to 1.3 in 2020.

### Age-stratified number of stroke events, 2011-2020

Table 3 provides an overview of stroke incidence trends from 2011 to 2020, categorized by age groups. Among individuals under 20 years of age, the total number of stroke incidence events showed a decrease from 579 in 2011 to 349 in 2020. Specifically, first stroke events decreased from 520 in 2011 to 312 in 2020, while recurrent cases dropped from 59 in 2011 to 37 in 2020. For those in the 20-29 age group, there was a decline in the total number of stroke events, from 583 in 2011 to 540 in 2020. First stroke events decreased from 527 in 2011 to 471 in 2020, whereas recurrences increased from 56 in 2011 to 69 in 2020. In the age group of 30-39, the number declined from 2,144 to 1,846. A similar decreasing trend was observed in the 40-49 age group, where stroke events decreased from 7,775 to 6,230. Similarly, the 50-59 age group experienced a decline from 16,795 to 16,102, and the 60-69 age group showed a decrease from 21,541 to 24,240. Among those aged 70-79, the total number of stroke events reduced from 32,061 in 2012 to 28,143 in 2020. For individuals aged 80 and above, there was a decrease from 19,035 incidents in 2011 to 29,177 in 2020. Overall, increasing trends were observed across total, first, and recurrent stroke events, with prominence in the age groups of 60-69 and those aged 80 and above.

The age-stratified data was further subdivided by sex, as shown in Supplementary Materials 1 and 2. Among males, there was an increase in the number of stroke incidence events in the age groups of 50-59, 60-69, 70-79, and 80 and above from 2011 to 2020. Additionally, with each advancing age group, there was a corresponding rise in the incidence of total, first, and recurrent strokes. For females, there was an increasing trend in the number of stroke incidence events observed in the age group of 80 and above from 2011 to 2020.

### Incidence rate of stroke, 2011-2020

**Figure 1.**
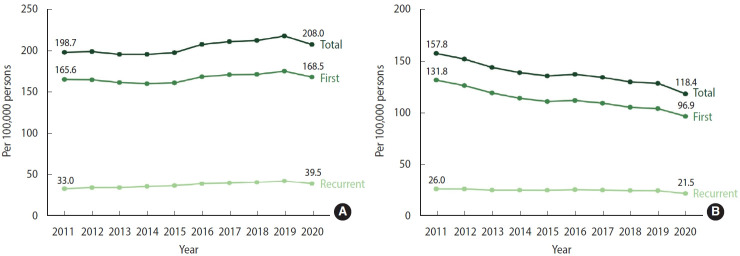
Incidence rate of stroke, 2011-2020. (A) Crude. (B) Age-standardized.

Between 2011 and 2015, the overall crude incidence rate of stroke (per 100,000 person-years) remained relatively stable at around 200. However, from 2016 to 2019, the rate experienced an increase, reaching a peak of 218.4, before slightly declining to 208.0 in 2020 (Figure 1A, Supplementary Material 3). The incidence rate of first stroke followed a similar trend to the total stroke events. During the same period, the incidence of recurrent stroke steadily increased from 2011 to 2019 and then showed a slight decrease in 2020. In contrast, the age-standardized incidence rate of stroke (per 100,000 person-years) exhibited a consistent decline, dropping from 157.8 in 2011 to 118.4 in 2020. The age-standardized incidence rates of total, first, and recurrent stroke displayed a similar trend to the overall incidence rate (Figure 1B, Supplementary Material 4).

**Figure 2.**
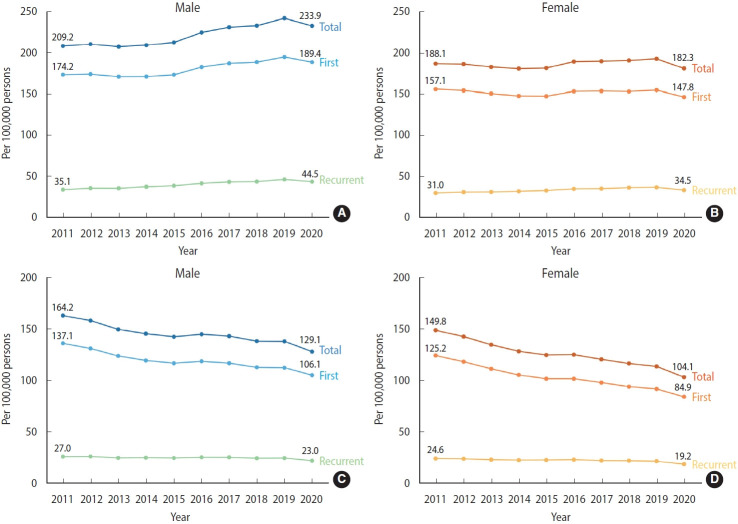
Sex-stratified incidence rate of stroke, 2011-2020. (A, B) Crude. (C, D) Age-standardized.

When examining the incidence rates by sex, the crude incidence rate in males remained stable from 2011 to 2015, but experienced an increase starting in 2016, reaching a peak of 242.9 per 100,000 person-years in 2019, and then slightly decreased in 2020 (Figure 2A, Supplementary Material 5). In females, the incidence rates for total, first, and recurrent stroke increased from 2011 to 2019 and then decreased in 2020 (Figure 2B, Supplementary Material 5). The age-standardized incidence rates for both total and first stroke consistently decreased from 2011 to 2020 for both males and females. However, the age-standardized rate of recurrent stroke displayed similar trends between the sexes. In both males and females, the rate maintained relative stability from 2011 to 2019, with a slight decrease observed in 2020 (Figure 2C and D, Supplementary Material 6).

### Case fatality rate of stroke, 2011-2020

**Figure 3.**
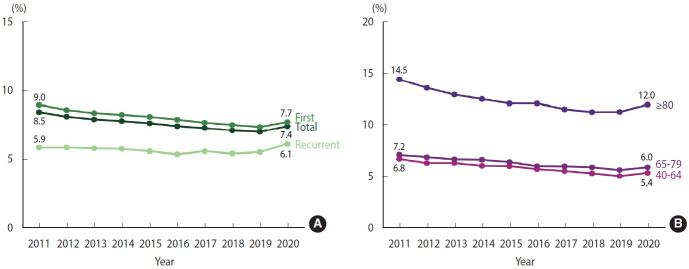
30-Day case fatality rate of stroke, 2011-2020. (A) Crude. (B) Age-stratified.

From 2011 to 2019, there was a reduction in the 30-day case fatality rates for both total stroke and first stroke, with rates decreasing from 8.5% to 7.4% and 9.0% to 7.7%, respectively. However, in 2020, both measures showed a slight upward trend. In the case of recurrent stroke, the 30-day case fatality rate remained relatively stable from 2011 to 2019 but showed an increase in 2020, reaching a peak of 6.1% (Figure 3A, Supplementary Material 7). When we further stratified the analysis into 3 age groups, we observed similar patterns across all categories. For stroke patients in the 40-64, 65-79, and ≥ 80 age groups, there was a consistent decline in the 30-day case fatality rates from 2011 to 2019. However, a reversal of this trend, marking an increase, was observed in 2020 (Figure 3B, Supplementary Material 8).

**Figure 4.**
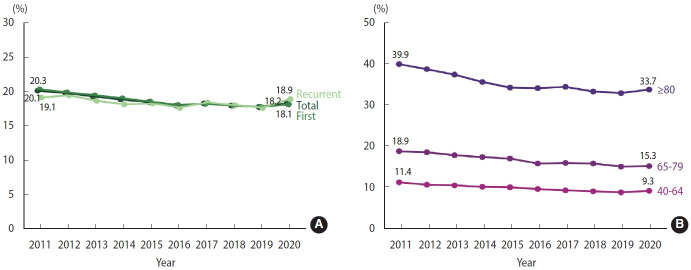
1-Year case fatality rate of stroke, 2011-2020. (A) Crude. (B) Age-stratified.

The 1-year case fatality rates for both total stroke and first stroke declined from around 20% in 2011 to approximately 18% in 2016, after which the rates remained stable until 2019, then slightly increased to about 18% in 2020. The 1-year case fatality rate for recurrent stroke displayed fluctuating trends, with an overall decline from 2011 to 2019, followed by an increase to 18.9% in 2020 (Figure 4A, Supplementary Material 9). These trends were evident across all age groups from 2011 to 2019, suggesting that factors other than age-structure changes impacted the 1-year case fatality rate. When the data were stratified into 3 age groups, a higher 1-year case fatality rate for stroke was observed in the older cohorts. Despite an overall downward trend from 2011 to 2019 across all age groups, the 1-year case fatality rate for stroke saw an upturn in 2020 (Figure 4B, Supplementary Material 10).

### Positive predictive values of the identification algorithms for stroke events

The PPVs of the algorithms used to identify initial and recurrent stroke events across different hospital types are outlined in Supplementary Material 11. The algorithm for identifying first stroke events demonstrated a PPV of 83.4% in tertiary hospitals, 94.1% in secondary hospitals, and 70.2% in primary hospitals. When fatal events where the cause of death was recorded as stroke were considered as positive events, the PPV increased to 70.7% in primary hospitals, while remaining unchanged in secondary and tertiary hospitals. The crude pooled PPV was 82.3%. When weighted according to the medical institution first visited and the highest-level medical institution during the episode, it was 88.2% and 88.1%, respectively.

The recurrent stroke identification algorithm exhibited a PPV of 75.7% in tertiary hospitals, 89.0% in secondary hospitals, and 63.3% in primary hospitals. When stroke-related fatalities were considered as positive events, the PPV increased to 65.6% in primary hospitals, with no changes observed in tertiary and secondary hospitals. The crude pooled PPV of the recurrent stroke identification algorithm was 78.4%, and the weighted pooled PPV according to medical institution first visited and the highest-level medical institution during the episode stood at 80.8% and 81.0%, respectively.

## DISCUSSION

This study provides a detailed examination of the decade-long trends in the incidence and case fatality rates of stroke in Korea. The crude overall incidence rate of stroke was observed to remain steady from 2011 to 2015, subsequently increase from 2016 to 2019, and then slightly decline in 2020. However, the age-standardized incidence rate exhibited a consistent decrease throughout the study period. When stratified by sex, the crude incidence rate in males showed an upward trend, whereas in females it remained relatively stable. The case fatality rates for both total and first stroke events decreased until 2016, remained stable until 2019, and then increased in 2020. Notably, the 1-year case fatality rates were significantly higher in older age groups. The potential impact of the coronavirus disease 2019 (COVID-19) pandemic in 2020 on healthcare accessibility, including access to medical systems, as well as its broader societal and cultural implications, necessitates a thoughtful and cautious interpretation of its effects on the accuracy of the identification algorithms.

In 2018, the Korean Stroke Society released an executive summary of stroke statistics in Korea, which was based on data obtained from the NHIS database [12]. The study found that the age-standardized and sex-standardized incidence rate of first stroke in Korea decreased from 105.8 (per 100,000 person-years) in 2007 to 92.2 (per 100,000 person-years) in 2013. The study also employed a stroke identification algorithm for claims data, which was developed and validated using a multicenter stroke registry database [13]. This algorithm selected 17 key identifiers from the claims data and established 37 conditions through combinations of these key identifiers. While this algorithm aimed to maximize the distinction between true and false acute stroke events, it required a larger amount of clinical data and might be more susceptible to changes in treatment patterns and health insurance policies. In contrast, our study utilized a more practical algorithm to monitor the annual occurrence trends of stroke over the long term while maintaining an acceptable PPV. Another Korean study analyzed NHIS data and reported that the stroke incidence rate among individuals with disabilities was 2.5 times higher than that among non-disabled individuals [14]. However, in this study, stroke was defined solely based on diagnostic codes. Other countries have also conducted studies on the incidence rate of stroke. In Japan, studies on stroke incidence utilized various methods, including registration systems at the prefecture or city level, community cohort studies, and medical institution network studies. These studies followed standardized diagnostic criteria, such as the WHO-MONICA criteria or case report forms to accurately document and diagnose strokes [15,16]. For instance, when comparing stroke incidence between urban and rural communities in Japan, significant declines in age-standardized incidence rates were observed [15]. In urban communities, the incidence decreased from 6.60 to 1.15 (per 1,000 person-years) for males and 3.28 to 0.59 for females. Similarly, in rural communities, the corresponding incidence decreased from 11.51 to 1.98 for males and 6.46 to 1.31 for females. In Takashima City, the age-standardized and sex-standardized incidence rates of stroke (per 100,000 person-years) decreased from 327 in the early period of the study (1990 to 2001) to 206 in the later period (2002 to 2010) [16]. In Taiwan, stroke incidence rates were also monitored using the NHI Research Database. Here, new stroke incidence was defined based on whether imaging tests such as computed tomography or magnetic resonance imaging were conducted during the acute treatment period [17]. Between 2004 and 2011, the age-adjusted incidence of all strokes (per 100,000 person-years) in Taiwan decreased by 16%, from 251 to 210. Overall, these findings suggest that both Japan and Taiwan have seen significant declines in stroke incidence over the years; however, the absence of an established system for producing disease statistics makes it difficult to update statistics consistently every year.

This study has several strengths that contribute to its robustness. First, it is a nationwide study that offers a comprehensive examination of stroke incidence and case fatality rate trends over a 10-year period in Korea. The use of a large database, the NHIS, which covers approximately 97% of the Korean population, ensured a representative sample. Moreover, the study analyzed trends based on sex and age groups, providing a detailed understanding of variations in stroke incidence and case fatality rates among different population segments. A notable feature of this study is the production of crucial data concerning recurrent stroke events. This emphasizes the importance of strategies aimed at preventing recurrent strokes and efficiently managing stroke risk factors, with the ultimate objective of reducing the overall societal and individual burden associated with stroke.

However, it is important to acknowledge the limitations of our study. First, we must consider that the COVID-19 pandemic in 2020 may have decreased the accessibility of medical services, a significant factor that could impact the analysis results within our study. Second, special attention is needed when interpreting the fatality rates for individuals aged 80 and above. The increased mortality in this age group may be influenced by various complex factors or be attributed to natural causes associated with aging, implying that these fatality rates may not exclusively represent the direct outcomes of stroke incidence. Third, the use of health insurance claim data introduces the potential for overlooked stroke cases among individuals not seeking medical care or those experiencing stroke events outside the country. Changes in diagnostic tools and healthcare systems can also influence observed incidence rates. Complementary studies should focus on assessing the incidence of events within the community where medical care was not sought. Fourth, efforts were made to strengthen validity by incorporating information on diagnostic tests and interventional treatments alongside diagnosis codes. However, we were unable to access test results. To address this limitation, a validation study employing medical record investigations was conducted, revealing a PPV of 88.6%. While this value may not be entirely satisfactory, it is comparable to findings from other international studies and offers a usable level for monitoring stroke incidence rates. Fifth, it should be noted that the NHIS database does not contain information on drug prescriptions, examinations, or procedures not covered by the NHIS scheme. For instance, if a stroke patient undergoes brain imaging without health insurance reimbursement, the case may not be identified as a stroke event due to the absence of information on brain imaging in the database. Finally, this study did not consider the subtypes of stroke (ischemic vs. hemorrhagic). To accurately discern stroke subtypes, detailed clinical information and examination results are vital. Consequently, hospital-based registry studies are necessary. These studies would enable the comprehensive collection of clinical data and diagnostic information, thereby paving the way for a more granular analysis of stroke subtypes.

## CONCLUSION

This nationwide study offers crucial insights into the trends of stroke incidence and case fatality rates in Korea, and its strengths include the use of a large-scale database and the analysis of trends segmented by sex and age groups. Nonetheless, it is essential to recognize the limitations inherent to the use of health insurance claims data, the necessity for community-focused studies, certain data usage restrictions, and the requirement for hospital-based registry studies to accurately determine stroke subtypes. While our findings depict a decade-long downward trend in stroke incidence, stroke continues to be a significant public health concern in Korea, contributing substantially to fatality and disability rates. This study serves as a valuable resource for clinicians, researchers, healthcare policymakers, and the public. By providing comprehensive national data on stroke, it aids in the creation of healthcare plans and paves the way for future research, ultimately enhancing the prevention, management, and treatment of cardiovascular diseases.

## Figures and Tables

**Figure f5-epih-46-e2024003:**
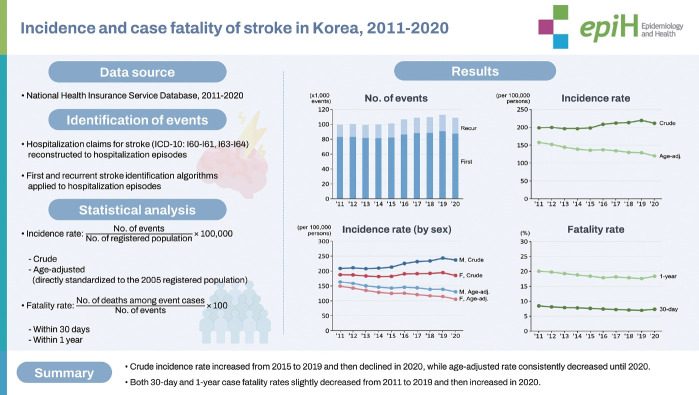


**Table 1.** Identification algorithms for first and recurrent stroke events

Event	Diagnosis codes (ICD-10)	Identification algorithm
First	Primary, I63-I64 (+)	(Brain imaging and [episode length ≥3 days or death]) or therapeutic intervention^1^ or death
	Primary, I63-I64 (-); All, I60-I61 (+)	(Brain imaging and [episode length ≥3 days or death]) or therapeutic intervention^1^ or death
	Primary, I63-I64 (-); All, I60-I61 (-); Secondary or lower, I63-I64 (+)	Therapeutic intervention^1^ and (episode length ≥3 days or death)
Recurrent	Primary, I63-I64 (+)	(Brain imaging or therapeutic intervention^1^) and (episode length ≥3 days or death
	Primary, I63-I64 (-); Primary, I60-I61 (+)	(Brain imaging or therapeutic intervention^1^) and (episode length ≥3 days or death)
	Primary, I60-I61, I63-I64 (-); Secondary or lower, I60-I61, I63-I64 (+)	Therapeutic intervention^1^ and (episode length ≥3 days or death)

ICD-10, International Classification of Disease, 10th revision.

1 Including intravenous thrombolysis, endovascular treatment, coil embolization, or other specific therapeutic interventions for stroke.

**Table 2.** Number of stroke events, 2011-2020

Sex	2011	2012	2013	2014	2015	2016	2017	2018	2019	2020
Both sexes										
Total	99,554	100,466	99,216	99,608	101,011	106,420	108,390	109,228	112,096	106,807
First	82,999	83,164	81,801	81,461	82,237	86,353	87,785	88,170	90,231	86,535
Recurrent	16,555	17,302	17,415	18,147	18,774	20,067	20,605	21,058	21,865	20,272
Male										
Total	52,483	53,284	52,692	53,389	54,418	57,679	59,382	59,921	62,217	59,887
First	43,683	44,029	43,427	43,613	44,310	46,817	48,058	48,494	50,093	48,487
Recurrent	8,800	9,255	9,265	9,776	10,108	10,862	11,324	11,427	12,124	11,400
Female										
Total	47,071	47,182	46,524	46,219	46,593	48,741	49,008	49,307	49,879	46,920
First	39,316	39,135	38,374	37,848	37,927	39,536	39,727	39,676	40,138	38,048
Recurrent	7,755	8,047	8,150	8,371	8,666	9,205	9,281	9,631	9,741	8,872

**Table 3.** Age-stratified number of stroke events, 2011-2020

Age (yr)	2011	2012	2013	2014	2015	2016	2017	2018	2019	2020
Total										
<20	579	537	518	501	471	426	420	393	381	349
20-29	583	548	549	596	533	577	551	596	571	540
30-39	2,144	2,146	2,043	2,003	2,032	2,155	2,104	2,004	2,013	1,846
40-49	7,775	7,700	7,469	7,419	7,447	7,460	7,556	7,133	6,914	6,230
50-59	16,795	16,910	16,760	16,689	16,833	18,012	17,645	17,443	17,342	16,102
60-69	21,541	20,484	19,877	19,933	21,102	22,122	22,535	23,136	24,444	24,420
70-79	31,102	32,061	31,505	30,697	29,903	30,422	30,741	30,437	30,381	28,143
≥80	19,035	20,080	20,495	21,770	22,690	25,246	26,838	28,086	30,050	29,177
First										
<20	520	494	484	461	443	388	384	362	336	312
20-29	527	493	495	534	462	509	480	517	500	471
30-39	1,945	1,912	1,826	1,788	1,786	1,923	1,841	1,772	1,789	1,632
40-49	6,839	6,713	6,514	6,437	6,462	6,467	6,547	6,103	5,967	5,459
50-59	14,269	14,310	14,101	14,004	14,081	14,940	14,690	14,435	14,444	13,584
60-69	17,640	16,653	16,240	16,159	16,956	17,795	18,101	18,598	19,568	19,744
70-79	25,266	25,889	25,234	24,338	23,584	23,951	24,093	23,896	23,629	22,132
≥80	15,993	16,700	16,907	17,740	18,463	20,380	21,649	22,487	23,998	23,201
Recurrent										
<20	59	43	34	40	28	38	36	31	45	37
20-29	56	55	54	62	71	68	71	79	71	69
30-39	199	234	217	215	246	232	263	232	224	214
40-49	936	987	955	982	985	993	1,009	1,030	947	771
50-59	2,526	2,600	2,659	2,685	2,752	3,072	2,955	3,008	2,898	2,518
60-69	3,901	3,831	3,637	3,774	4,146	4,327	4,434	4,538	4,876	4,676
70-79	5,836	6,172	6,271	6,359	6,319	6,471	6,648	6,541	6,752	6,011
≥80	3,042	3,380	3,588	4,030	4,227	4,866	5,189	5,599	6,052	5,976

## Supplementary materials

Supplementary Material 1. Age-stratified incidence stroke event in males, 2011-2020

Supplementary Material 2. Age-stratified incidence stroke event in females, 2011-2020

Supplementary Material 3. Crude incidence rate of stroke per 100,000 person-years in 2011-2020

Supplementary Material 4. Age-adjusted incidence rate of stroke per 100,000 person-years in 2011-2020

Supplementary Material 5. Crude sex-specific incidence rate of stroke per 100,000 person-years in 2011-2020

Supplementary Material 6. Age-adjusted and sex-specific incidence rate of stroke per 100,000 person-years in 2011-2020

Supplementary Material 7. Thirty-day case fatality of stroke, 2011-2020 (%)

Supplementary Material 8. Age-stratified thirty-day case fatality of stroke, 2011-2020 (%)

Supplementary Material 9. One-year case fatality of stroke, 2011-2020 (%)

Supplementary Material 10. Age-stratified one-year case fatality of stroke, 2011-2020 (%)

Supplementary Material 11. Positive predictive value of the working definition for stroke event
