# Supplementary material for: Incidence and case fatality of stroke in Korea, 2011-2020
Source: Epidemiol Health. 2023 Dec 26;46:e2024003. doi: 10.4178/epih.e2024003 (PMC10928468; doi:10.4178/epih.e2024003)
Supplement: Supplementary Material 1. — Age-stratified incidence stroke event in males, 2011-2020 [file epih-46-e2024003-Supplementary-1.docx]

**Table of Contents**

[**Supplementary Material 1.** Age-stratified incidence stroke event in males, 2011-2020 2](#_Toc156248264)

[**Supplementary Material 2.** Age-stratified incidence stroke event in females, 2011-2020 3](#_Toc156248265)

[**Supplementary Material 3.** Crude incidence rate of stroke per 100,000 person-years in 2011-2020 4](#_Toc156248266)

[**Supplementary Material 4.** Age-adjusted incidence rate of stroke per 100,000 person-years in 2011-2020 5](#_Toc156248267)

[**Supplementary Material 5.** Crude sex-specific incidence rate of stroke per 100,000 person-years in 2011-2020 6](#_Toc156248268)

[**Supplementary Material 6.** Age-adjusted and sex-specific incidence rate of stroke per 100,000 person-years in 2011-2020 7](#_Toc156248269)

[**Supplementary Material 7.** Thirty-day case fatality of stroke, 2011-2020 (%) 8](#_Toc156248270)

[**Supplementary Material 8.** Age-stratified thirty-day case fatality of stroke, 2011-2020 (%) 9](#_Toc156248271)

[**Supplementary Material 9.** One-year case fatality of stroke, 2011-2020 (%) 10](#_Toc156248272)

[**Supplementary Material 10.** Age-stratified one-year case fatality of stroke, 2011-2020 (%) 11](#_Toc156248273)

[**Supplementary Material 11.** Positive predictive value of the working definition for stroke event 12](#_Toc156248274)

Supplementary Material 1. Age-stratified incidence stroke event in males, 2011-2020

| **Male** | | **Year** | | | | | | | | | |
| --- | --- | --- | --- | --- | --- | --- | --- | --- | --- | --- | --- |
|  |  | **2011** | **2012** | **2013** | **2014** | **2015** | **2016** | **2017** | **2018** | **2019** | **2020** |
| **Total** | | | | | | | | | | | |
|  | <20 | 341 | 336 | 315 | 285 | 260 | 235 | 245 | 220 | 213 | 190 |
|  | 20-29 | 350 | 335 | 341 | 370 | 297 | 333 | 303 | 340 | 334 | 313 |
|  | 30-39 | 1,423 | 1,394 | 1,309 | 1,288 | 1,327 | 1,410 | 1,384 | 1,246 | 1,312 | 1,183 |
|  | 40-49 | 5,159 | 5,134 | 4,941 | 4,865 | 4,937 | 4,992 | 5,055 | 4,745 | 4,503 | 4,079 |
|  | 50-59 | 11,122 | 11,271 | 11,241 | 11,295 | 11,401 | 12,214 | 12,078 | 11,797 | 12,007 | 11,213 |
|  | 60-69 | 12,965 | 12,574 | 12,287 | 12,611 | 13,227 | 14,091 | 14,598 | 15,262 | 16,130 | 16,320 |
|  | 70-79 | 14,757 | 15,347 | 15,270 | 15,193 | 14,924 | 15,487 | 15,944 | 15,945 | 16,323 | 15,462 |
|  | ≥80 | 6,366 | 6,893 | 6,988 | 7,482 | 8,045 | 8,917 | 9,775 | 10,366 | 11,395 | 11,127 |
| **First** | | | | | | | | | | | |
|  | <20 | 308 | 313 | 293 | 259 | 247 | 218 | 225 | 202 | 190 | 173 |
|  | 20-29 | 311 | 303 | 306 | 325 | 261 | 294 | 264 | 290 | 290 | 272 |
|  | 30-39 | 1,292 | 1,260 | 1,185 | 1,169 | 1,185 | 1,280 | 1,227 | 1,126 | 1,178 | 1,060 |
|  | 40-49 | 4,582 | 4,488 | 4,320 | 4,252 | 4,312 | 4,358 | 4,420 | 4,093 | 3,948 | 3,615 |
|  | 50-59 | 9,445 | 9,576 | 9,496 | 9,521 | 9,566 | 10,164 | 10,141 | 9,858 | 10,089 | 9,522 |
|  | 60-69 | 10,586 | 10,147 | 10,006 | 10,145 | 10,613 | 11,343 | 11,691 | 12,273 | 12,892 | 13,154 |
|  | 70-79 | 11,941 | 12,354 | 12,196 | 12,005 | 11,736 | 12,079 | 12,320 | 12,440 | 12,586 | 12,023 |
|  | ≥80 | 5,218 | 5,588 | 5,625 | 5,937 | 6,390 | 7,081 | 7,770 | 8,212 | 8,920 | 8,668 |
| **Recurrent** | | | | | | | | | | | |
|  | <20 | 33 | 23 | 22 | 26 | 13 | 17 | 20 | 18 | 23 | 17 |
|  | 20-29 | 39 | 32 | 35 | 45 | 36 | 39 | 39 | 50 | 44 | 41 |
|  | 30-39 | 131 | 134 | 124 | 119 | 142 | 130 | 157 | 120 | 134 | 123 |
|  | 40-49 | 577 | 646 | 621 | 613 | 625 | 634 | 635 | 652 | 555 | 464 |
|  | 50-59 | 1,677 | 1,695 | 1,745 | 1,774 | 1,835 | 2,050 | 1,937 | 1,939 | 1,918 | 1,691 |
|  | 60-69 | 2,379 | 2,427 | 2,281 | 2,466 | 2,614 | 2,748 | 2,907 | 2,989 | 3,238 | 3,166 |
|  | 70-79 | 2,816 | 2,993 | 3,074 | 3,188 | 3,188 | 3,408 | 3,624 | 3,505 | 3,737 | 3,439 |
|  | ≥80 | 1,148 | 1,305 | 1,363 | 1,545 | 1,655 | 1,836 | 2,005 | 2,154 | 2,475 | 2,459 |
