# Supplementary material for: Incidence and case fatality of stroke in Korea, 2011-2020
Source: Epidemiol Health. 2023 Dec 26;46:e2024003. doi: 10.4178/epih.e2024003 (PMC10928468; doi:10.4178/epih.e2024003)
Supplement: Supplementary Material 2. — Age-stratified incidence stroke event in females, 2011-2020 [file epih-46-e2024003-Supplementary-2.docx]

Supplementary Material 2. Age-stratified incidence stroke event in females, 2011-2020

| **Female** | | **Year** | | | | | | | | | |
| --- | --- | --- | --- | --- | --- | --- | --- | --- | --- | --- | --- |
|  |  | **2011** | **2012** | **2013** | **2014** | **2015** | **2016** | **2017** | **2018** | **2019** | **2020** |
| **Total** | | | | | | | | | | | |
|  | <20 | 238 | 201 | 203 | 216 | 211 | 191 | 175 | 173 | 168 | 159 |
|  | 20-29 | 233 | 213 | 208 | 226 | 236 | 244 | 248 | 256 | 237 | 227 |
|  | 30-39 | 721 | 752 | 734 | 715 | 705 | 745 | 720 | 758 | 701 | 663 |
|  | 40-49 | 2,616 | 2,566 | 2,528 | 2,554 | 2,510 | 2,468 | 2,501 | 2,388 | 2,411 | 2,151 |
|  | 50-59 | 5,673 | 5,639 | 5,519 | 5,394 | 5,432 | 5,798 | 5,567 | 5,646 | 5,335 | 4,889 |
|  | 60-69 | 8,576 | 7,910 | 7,590 | 7,322 | 7,875 | 8,031 | 7,937 | 7,874 | 8,314 | 8,100 |
|  | 70-79 | 16,345 | 16,714 | 16,235 | 15,504 | 14,979 | 14,935 | 14,797 | 14,492 | 14,058 | 12,681 |
|  | ≥80 | 12,669 | 13,187 | 13,507 | 14,288 | 14,645 | 16,329 | 17,063 | 17,720 | 18,655 | 18,050 |
| **First** | | | | | | | | | | | |
|  | <20 | 212 | 181 | 191 | 202 | 196 | 170 | 159 | 160 | 146 | 139 |
|  | 20-29 | 216 | 190 | 189 | 209 | 201 | 215 | 216 | 227 | 210 | 199 |
|  | 30-39 | 653 | 652 | 641 | 619 | 601 | 643 | 614 | 646 | 611 | 572 |
|  | 40-49 | 2,257 | 2,225 | 2,194 | 2,185 | 2,150 | 2,109 | 2,127 | 2,010 | 2,019 | 1,844 |
|  | 50-59 | 4,824 | 4,734 | 4,605 | 4,483 | 4,515 | 4,776 | 4,549 | 4,577 | 4,355 | 4,062 |
|  | 60-69 | 7,054 | 6,506 | 6,234 | 6,014 | 6,343 | 6,452 | 6,410 | 6,325 | 6,676 | 6,590 |
|  | 70-79 | 13,325 | 13,535 | 13,038 | 12,333 | 11,848 | 11,872 | 11,773 | 11,456 | 11,043 | 10,109 |
|  | ≥80 | 10,775 | 11,112 | 11,282 | 11,803 | 12,073 | 13,299 | 13,879 | 14,275 | 15,078 | 14,533 |
| **Recurrent** | | | | | | | | | | | |
|  | <20 | 26 | 20 | 12 | 14 | 15 | 21 | 16 | 13 | 22 | 20 |
|  | 20-29 | 17 | 23 | 19 | 17 | 35 | 29 | 32 | 29 | 27 | 28 |
|  | 30-39 | 68 | 100 | 93 | 96 | 104 | 102 | 106 | 112 | 90 | 91 |
|  | 40-49 | 359 | 341 | 334 | 369 | 360 | 359 | 374 | 378 | 392 | 307 |
|  | 50-59 | 849 | 905 | 914 | 911 | 917 | 1,022 | 1,018 | 1,069 | 980 | 827 |
|  | 60-69 | 1,522 | 1,404 | 1,356 | 1,308 | 1,532 | 1,579 | 1,527 | 1,549 | 1,638 | 1,510 |
|  | 70-79 | 3,020 | 3,179 | 3,197 | 3,171 | 3,131 | 3,063 | 3,024 | 3,036 | 3,015 | 2,572 |
|  | ≥80 | 1,894 | 2,075 | 2,225 | 2,485 | 2,572 | 3,030 | 3,184 | 3,445 | 3,577 | 3,517 |
