# Supplementary material for: Incidence and case fatality of stroke in Korea, 2011-2020
Source: Epidemiol Health. 2023 Dec 26;46:e2024003. doi: 10.4178/epih.e2024003 (PMC10928468; doi:10.4178/epih.e2024003)
Supplement: Supplementary Material 3. — Crude incidence rate of stroke per 100,000 person-years in 2011-2020 [file epih-46-e2024003-Supplementary-3.docx]

Supplementary Material 3. Crude incidence rate of stroke per 100,000 person-years in 2011-2020

| **Characteristics**  **of stroke** | **Year** | | | | | | | | | |
| --- | --- | --- | --- | --- | --- | --- | --- | --- | --- | --- |
|  | **2011** | **2012** | **2013** | **2014** | **2015** | **2016** | **2017** | **2018** | **2019** | **2020** |
| Total | 198.7 | 199.6 | 196.2 | 196.2 | 198.2 | 208.2 | 211.6 | 212.9 | 218.4 | 208.0 |
| First | 165.6 | 165.2 | 161.8 | 160.5 | 161.4 | 168.9 | 171.4 | 171.9 | 175.8 | 168.5 |
| Recurrent | 33.0 | 34.4 | 34.4 | 35.7 | 36.8 | 39.3 | 40.2 | 41.0 | 42.6 | 39.5 |
