# Supplementary material for: Incidence and case fatality of stroke in Korea, 2011-2020
Source: Epidemiol Health. 2023 Dec 26;46:e2024003. doi: 10.4178/epih.e2024003 (PMC10928468; doi:10.4178/epih.e2024003)
Supplement: Supplementary Material 4. — Age-adjusted incidence rate of stroke per 100,000 person-years in 2011-2020 [file epih-46-e2024003-Supplementary-4.docx]

Supplementary Material 4. Age-adjusted incidence rate of stroke per 100,000 person-years in 2011-2020

| **Characteristics**  **of stroke** | **Year** | | | | | | | | | |
| --- | --- | --- | --- | --- | --- | --- | --- | --- | --- | --- |
|  | **2011** | **2012** | **2013** | **2014** | **2015** | **2016** | **2017** | **2018** | **2019** | **2020** |
| Total | 157.8 | 152.4 | 144.2 | 139.1 | 135.8 | 137.4 | 134.4 | 130.0 | 128.6 | 118.4 |
| First | 131.8 | 126.4 | 119.3 | 114.2 | 111.1 | 112.1 | 109.5 | 105.6 | 104.3 | 96.9 |
| Recurrent | 26.0 | 26.0 | 24.9 | 24.8 | 24.7 | 25.3 | 24.9 | 24.4 | 24.3 | 21.5 |
