# Supplementary material for: Incidence and case fatality of stroke in Korea, 2011-2020
Source: Epidemiol Health. 2023 Dec 26;46:e2024003. doi: 10.4178/epih.e2024003 (PMC10928468; doi:10.4178/epih.e2024003)
Supplement: Supplementary Material 5. — Crude sex-specific incidence rate of stroke per 100,000 person-years in 2011-2020 [file epih-46-e2024003-Supplementary-5.docx]

Supplementary Material 5. Crude sex-specific incidence rate of stroke per 100,000 person-years in 2011-2020

| **Sex** | **Year** | | | | | | | | | |
| --- | --- | --- | --- | --- | --- | --- | --- | --- | --- | --- |
|  | **2011** | **2012** | **2013** | **2014** | **2015** | **2016** | **2017** | **2018** | **2019** | **2020** |
| **Male** |  |  |  |  |  |  |  |  |  |  |
| Total | 209.2 | 211.5 | 208.4 | 210.4 | 213.8 | 225.9 | 232.2 | 234.0 | 242.9 | 233.9 |
| First | 174.2 | 174.8 | 171.8 | 171.9 | 174.1 | 183.4 | 187.9 | 189.4 | 195.6 | 189.4 |
| Recurrent | 35.1 | 36.7 | 36.6 | 38.5 | 39.7 | 42.5 | 44.3 | 44.6 | 47.3 | 44.5 |
| **Female** |  |  |  |  |  |  |  |  |  |  |
| Total | 188.1 | 187.5 | 184.1 | 182.0 | 182.8 | 190.5 | 191.0 | 191.9 | 193.9 | 182.3 |
| First | 157.1 | 155.6 | 151.8 | 149.1 | 148.8 | 154.5 | 154.9 | 154.4 | 156.0 | 147.8 |
| Recurrent | 31.0 | 32.0 | 32.2 | 33.0 | 34.0 | 36.0 | 36.2 | 37.5 | 37.9 | 34.5 |
