# Supplementary material for: Incidence and case fatality of stroke in Korea, 2011-2020
Source: Epidemiol Health. 2023 Dec 26;46:e2024003. doi: 10.4178/epih.e2024003 (PMC10928468; doi:10.4178/epih.e2024003)
Supplement: Supplementary Material 6. — Age-adjusted and sex-specific incidence rate of stroke per 100,000 person-years in 2011-2020 [file epih-46-e2024003-Supplementary-6.docx]

Supplementary Material 6. Age-adjusted and sex-specific incidence rate of stroke per 100,000 person-years in 2011-2020

| **Sex** | **Year** | | | | | | | | | |
| --- | --- | --- | --- | --- | --- | --- | --- | --- | --- | --- |
|  | **2011** | **2012** | **2013** | **2014** | **2015** | **2016** | **2017** | **2018** | **2019** | **2020** |
| **Male** |  |  |  |  |  |  |  |  |  |  |
| Total | 164.2 | 159.3 | 150.8 | 146.5 | 143.6 | 146.0 | 144.2 | 139.3 | 139.1 | 129.1 |
| First | 137.1 | 132.2 | 125.0 | 120.5 | 117.9 | 119.7 | 117.9 | 113.9 | 113.5 | 106.1 |
| Recurrent | 27.0 | 27.1 | 25.8 | 26.0 | 25.7 | 26.3 | 26.3 | 25.4 | 25.6 | 23.0 |
| **Female** |  |  |  |  |  |  |  |  |  |  |
| Total | 149.8 | 143.6 | 135.7 | 129.3 | 125.7 | 126.0 | 121.5 | 117.4 | 114.5 | 104.1 |
| First | 125.2 | 119.2 | 112.1 | 106.2 | 102.4 | 102.4 | 98.8 | 94.8 | 92.5 | 84.9 |
| Recurrent | 24.6 | 24.4 | 23.6 | 23.1 | 23.2 | 23.6 | 22.7 | 22.6 | 22.1 | 19.2 |
