# Supplementary material for: Incidence and case fatality of stroke in Korea, 2011-2020
Source: Epidemiol Health. 2023 Dec 26;46:e2024003. doi: 10.4178/epih.e2024003 (PMC10928468; doi:10.4178/epih.e2024003)
Supplement: Supplementary Material 7. — Thirty-day case fatality of stroke, 2011-2020 (%) [file epih-46-e2024003-Supplementary-7.docx]

Supplementary Material 7. Thirty-day case fatality of stroke, 2011-2020 (%)

| **Characteristics**  **of stroke** | **Year** | | | | | | | | | |
| --- | --- | --- | --- | --- | --- | --- | --- | --- | --- | --- |
|  | **2011** | **2012** | **2013** | **2014** | **2015** | **2016** | **2017** | **2018** | **2019** | **2020** |
| Total | 8.5 | 8.1 | 7.9 | 7.8 | 7.7 | 7.4 | 7.3 | 7.1 | 7.0 | 7.4 |
| First | 9.0 | 8.6 | 8.4 | 8.3 | 8.1 | 7.9 | 7.7 | 7.5 | 7.4 | 7.7 |
| Recurrent | 5.9 | 5.9 | 5.8 | 5.8 | 5.6 | 5.4 | 5.6 | 5.4 | 5.6 | 6.1 |
