# Supplementary material for: Incidence and case fatality of stroke in Korea, 2011-2020
Source: Epidemiol Health. 2023 Dec 26;46:e2024003. doi: 10.4178/epih.e2024003 (PMC10928468; doi:10.4178/epih.e2024003)
Supplement: Supplementary Material 8. — Age-stratified thirty-day case fatality of stroke, 2011-2020 (%) [file epih-46-e2024003-Supplementary-8.docx]

Supplementary Material 8. Age-stratified thirty-day case fatality of stroke, 2011-2020 (%)

| **Age, years** | **Year** | | | | | | | | | |
| --- | --- | --- | --- | --- | --- | --- | --- | --- | --- | --- |
|  | **2011** | **2012** | **2013** | **2014** | **2015** | **2016** | **2017** | **2018** | **2019** | **2020** |
| > 80 | 14.5 | 13.7 | 13.0 | 12.6 | 12.2 | 12.2 | 11.6 | 11.3 | 11.3 | 12.0 |
| 65-79 | 7.2 | 6.9 | 6.7 | 6.7 | 6.5 | 6.1 | 6.1 | 6.0 | 5.7 | 6.0 |
| 40-64 | 6.8 | 6.4 | 6.4 | 6.1 | 6.1 | 5.8 | 5.6 | 5.4 | 5.1 | 5.4 |
