# Supplementary material for: Incidence and case fatality of stroke in Korea, 2011-2020
Source: Epidemiol Health. 2023 Dec 26;46:e2024003. doi: 10.4178/epih.e2024003 (PMC10928468; doi:10.4178/epih.e2024003)
Supplement: Supplementary Material 9. — One-year case fatality of stroke, 2011-2020 (%) [file epih-46-e2024003-Supplementary-9.docx]

Supplementary Material 9. One-year case fatality of stroke, 2011-2020 (%)

| **Characteristics**  **of stroke** | **Year** | | | | | | | | | |
| --- | --- | --- | --- | --- | --- | --- | --- | --- | --- | --- |
|  | **2011** | **2012** | **2013** | **2014** | **2015** | **2016** | **2017** | **2018** | **2019** | **2020** |
| Total | 20.1 | 19.8 | 19.3 | 18.8 | 18.4 | 18.0 | 18.2 | 17.9 | 17.7 | 18.2 |
| First | 20.3 | 19.8 | 19.4 | 19.0 | 18.5 | 18.0 | 18.2 | 17.9 | 17.8 | 18.1 |
| Recurrent | 19.1 | 19.4 | 18.6 | 18.1 | 18.2 | 17.7 | 18.4 | 18.0 | 17.7 | 18.9 |
