# Supplementary material for: Incidence and case fatality of stroke in Korea, 2011-2020
Source: Epidemiol Health. 2023 Dec 26;46:e2024003. doi: 10.4178/epih.e2024003 (PMC10928468; doi:10.4178/epih.e2024003)
Supplement: Supplementary Material 10. — Age-stratified one-year case fatality of stroke, 2011-2020 (%) [file epih-46-e2024003-Supplementary-10.docx]

Supplementary Material 10. Age-stratified one-year case fatality of stroke, 2011-2020 (%)

| **Age, years** | **Year** | | | | | | | | | |
| --- | --- | --- | --- | --- | --- | --- | --- | --- | --- | --- |
|  | **2011** | **2012** | **2013** | **2014** | **2015** | **2016** | **2017** | **2018** | **2019** | **2020** |
| > 80 | 39.9 | 38.7 | 37.4 | 35.6 | 34.2 | 34.1 | 34.4 | 33.3 | 32.9 | 33.7 |
| 65-79 | 18.9 | 18.7 | 18.0 | 17.5 | 17.2 | 16.0 | 16.1 | 16.0 | 15.2 | 15.3 |
| 40-64 | 11.4 | 10.8 | 10.6 | 10.3 | 10.2 | 9.7 | 9.4 | 9.2 | 8.9 | 9.3 |
