# Supplementary material for: Incidence and case fatality of stroke in Korea, 2011-2020
Source: Epidemiol Health. 2023 Dec 26;46:e2024003. doi: 10.4178/epih.e2024003 (PMC10928468; doi:10.4178/epih.e2024003)
Supplement: Supplementary Material 11. — Positive predictive value of the working definition for stroke event [file epih-46-e2024003-Supplementary-11.docx]

Supplementary Material 11. Positive predictive value of the working definition for stroke event

| **Classification of Hospitals** | **Characteristics of stroke** | | | | | | |
| --- | --- | --- | --- | --- | --- | --- | --- |
|  | **First event** | | |  | **Recurrent event** | | |
|  | **Epidemiological determination** | **Identification algorithm** | **PPV** |  | **Epidemiological determination** | **Identification algorithm** | **PPV** |
| **Hospital types** |  |  |  |  |  |  |  |
| Tertiary hospitals | 458 | 549 | 83.4% |  | 137 | 181 | 75.7% |
| Secondary hospitals | 335 | 356 | 94.1% |  | 154 | 173 | 89.0% |
| Primary hospitals | 275 | 392 | 70.2% |  | 57 | 90 | 63.3% |
| **Total** |  |  |  |  |  |  |  |
| General | 1,068 | 1,297 | 82.3% |  | 348 | 444 | 78.4% |
| Weight 1** | - | - | 88.2% |  | - | - | 80.8% |
| Weight 2^†^ | - | - | 88.1% |  | - | - | 81.0% |
| **Weighted pooled based on the initial medical institution | | | | | | | |
| †Weighted pooled based on the highest-level medical institution | | | | | | | |
| *PPV, Positive Predictive Value | | | | | | | |
